# Development of Multi-concentration Cu:Ag Bimetallic Nanoparticles as a Promising Bactericidal for Antibiotic-Resistant Bacteria as Evaluated with Molecular Docking Study

**DOI:** 10.1186/s11671-021-03547-6

**Published:** 2021-05-22

**Authors:** Shumaila Mureed, Sadia Naz, Ali Haider, Ali Raza, Anwar Ul-Hamid, Junaid Haider, Muhammad Ikram, Rabia Ghaffar, Muneeb Irshad, Abdul Ghaffar, Aamer Saeed

**Affiliations:** 1 Solar Cell Applications Research Lab, Department of Physics, Government College University Lahore, Lahore, 54000 Punjab Pakistan; 2 Department of Physics, Government College University Lahore, Lahore, 54000 Punjab Pakistan; 3 Tianjin Institute of Industrial Biotechnology, Chinese Academy of Sciences, Tianjin, 300308 China; 4 Department of Clinical Medicine and Surgery, University of Veterinary and Animal Sciences, Lahore, 54000 Punjab Pakistan; 5 Department of Physics, Riphah Institute of Computing and Applied Sciences (RICAS), Riphah International University, 14 Ali Road, Lahore, Pakistan; 6 Core Research Facilities, King Fahd University of Petroleum & Minerals, Dhahran, 31261 Saudi Arabia; 7 Division of Science and Technology, Department of Botany, University of Education, Lahore, 54000 Pakistan; 8 Department of Physics, University of Engineering and Technology, Lahore, 54890 Pakistan; 9 Department of Chemistry, Quaid-I-Azam University, Islamabad, 45320 Pakistan

**Keywords:** Bimetallic, Antimicrobial, Docking, HR-TEM, Cu:ag

## Abstract

The present study is concerned with evaluating the influence of various concentrations of Ag within Cu:Ag bimetallic nanoparticles developed for use as a promising anti-bacterial agent against antibiotic-resistant bacteria. Here, Cu:Ag bimetallic nanoparticles with various concentration ratios (2.5, 5.0, 7.5, and 10 wt%) of Ag in fixed amount of Cu labeled as 1:0.025, 1:0.050, 1:0.075, and 1:0.1 were synthesized using co-precipitation method with ammonium hydroxide and deionized water as solvent, polyvinyl pyrrolidone as a capping agent, and sodium borohydride and ascorbic acid as reducing agents. These formulated products were characterized through a variety of techniques. XRD confirmed phase purity and detected the presence of distinct fcc structures belonging to Cu and Ag phases. FTIR spectroscopy confirmed the presence of vibrational modes corresponding to various functional groups and recorded characteristic peak emanating from the bimetallic. UV–visible spectroscopy revealed reduction in band gap with increasing Ag content. SEM and HR-TEM micrographs revealed spherical morphology of Ag-doped Cu bimetallic with small and large scale agglomerations. The samples exhibited varying dimensions and interlayer spacing. Bactericidal action of synthesized Cu:Ag bimetallic NPs depicted statistically significant (*P* < 0.05) inhibition zones recorded for various concentrations of Ag dopant against *Staphylococcus aureus *(*S. aureus*)*, Escherichia coli *(*E. coli*)*,* and *Acinetobacter baumannii *(*A. baumannii*) ranging from (0.85–2.8 mm), (0.55–1.95 mm) and (0.65–1.85 mm), respectively. Broadly, Cu:Ag bimetallic NPs were found to be more potent against gram-positive compared with gram-negative. Molecular docking study of Ag–Cu bimetallic NPs was performed against *β*-lactamase which is a key enzyme of cell wall biosynthetic pathway from both *S. aureus* (Binding score: − 4.981 kcal/mol) and *A. bauminnii* (Binding score: − 4.013 kcal/mol). Similarly, binding interaction analysis against FabI belonging to fatty acid biosynthetic pathway from *A. bauminnii* (Binding score: − 3.385 kcal/mol) and *S. aureus* (Binding score: − 3.012 kcal/mol) along with FabH from *E. coli* (Binding score: − 4.372 kcal/mol) was undertaken. These theoretical computations indicate Cu-Ag bimetallic NPs as possible inhibitor of selected enzymes. It is suggested that exploring in vitro inhibition potential of these materials may open new avenues for antibiotic discovery.

## Introduction

A variety of micro-organisms composed of bacteria, fungi, viruses, and parasites are present within the earth and its environment. These species cause complications in the production and use of medical equipment, healthcare merchandise, processed foods, water purification systems, and domestic sanitation products [1, 2]. Antibiotics are routinely employed by physicians to kill bacteria that cause illness in humans and animals. The disadvantage of frequent use of antibiotics is that it makes bacteria drug-resistant with time. Antibiotics also serve to reduce the number of ‘good’ bacteria present in the body, which fight against infections. Illnesses resulting from infections caused by antibiotic-resistant bacteria have become a major cause for concern in the field of medicine today. In this respect, many germs have been identified to be drug-resistant [3–7]. Novel efforts are under way to address the issue of drug-resistant bacteria and substitute current antimicrobial agents with more efficient and complementary therapies. In this regard, nanotechnology has rendered a substantial contribution to the production of nanomaterials such as metallic and metal oxide NPs (i.e., Ag, Cu, CuO, TiO_2_, SiO_2_, MgO, and ZnO) to fight an ever-increasing number of antimicrobial-resistant microorganisms. Among these, Cu and Ag NPs have shown encouraging antimicrobial properties [8–12].

In recent years, bimetallic NPs have been developed and used for various applications in the fields of chemistry, material science, biotechnology, and environmental protection. Bimetallic NPs containing copper (Cu) and silver (Ag) with a high fraction of surface atoms and large specific surface area have been widely studied [13]. These bimetallic NPs are of great interest due to their enhanced chemical, optical, catalytic, biological, plasmonic, and especially antimicrobial properties [14–20]. Ag ions can be reduced by ethanol under atmospheric conditions at 800 to 1000 °C to obtain silver NPs [21, 22]. Silver NPs possess good antimicrobial efficacy, therefore, it is used in the production of sunscreen creams and water treatment [23]. Cu NPs are fabricated by the reduction of copper sulfate with hydrazine in ethylene glycol under microwave irradiation, and can also be used as an antibacterial agent [24–26].

Metals such as Cu and Ag individually do not have promising optical, catalytic, and structural properties and cannot be converted into bimetallics. On the other hand, combining both metals (Cu:Ag) offers new opportunities to tune the structure and morphology of the resulting product for desired applications. Based upon its final structure, e.g., core–shell, dumb-bell structure, two-interface structure, randomly-mixed structure, or flower-shape structure, bimetallic NPs can exhibit a range of antimicrobial activity [27–31]. Various methods are available for the synthesis of bimetallic NPs including co-precipitation, sol–gel, hydrothermal, reduction, micro-emulsion, and polyol method [32–37].

In this study, Cu:Ag bimetallic NPs were synthesized through co-precipitation method using ammonium hydroxide and deionized water as solvent, polyvinyl pyrrolidone as a capping agent, and sodium borohydride and ascorbic acid as reducing agents. Four samples with various concentrations were prepared. With increasing concentrations of Ag in prepared bimetallic NPs, samples showed enhanced activity against bacteria *acinetobacter baumannii* that causes fever and nausea. The synthesized material assumed red appearance during rapid growth suggesting that antimicrobial activity was enhanced with increasing concentrations of Ag in bimetallic NPs. Furthermore, In silico predictions using molecular docking study were performed to identify the interaction pattern of Cu:Ag bimetallic NPs against *β*-lactamase enzyme of cell wall biosynthetic pathway alongside FabI and FabH enzymes of fatty acid biosynthetic pathway.

## Methods

The current study was aimed to synthesize various concentrations of Ag within Cu:Ag bimetallic nanoparticles through hydrothermal route to investigate the efficacy of antibacterial agent against antibiotic-resistant bacteria.

### Materials

Copper (II) chloride (CuCl_2 0.2_H_2_O, 98.9%), and silver nitrate (AgNO_3_) as precursors, polyvinyl pyrrolidone (PVP, an average molecular weight of 40,000) as capping agent, sodium borohydride (NaBH_4_, 99.9%) and L-Ascorbic acid (C_6_H_8_O_6_, 99.0%) as reducing agents and ammonium hydroxide (NH_4_OH) were used in the present study after acquiring them from Sigma Aldrich, USA.

### Synthesis of bimetallic Cu:Ag NPs

**Fig. 1:**
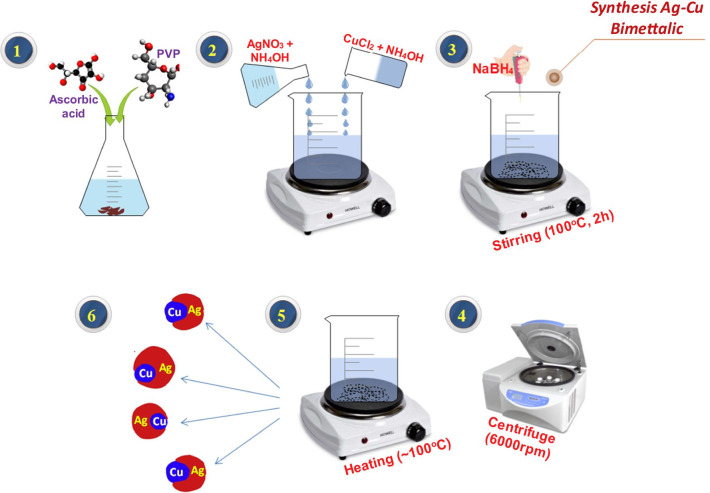
Illustration showing synthesis of Cu:Ag bimetallic NPs

Bimetallic Cu:Ag NPs were prepared using co-precipitation method as portrayed in Fig. 1. In deionized water, 1.25 g of PVP and 0.5 g of ascorbic acid were added and stirred vigorously at 100 °C. Two solutions of 40 mL of ammonium hydroxide were prepared individually; in one solution, 1.7 g copper chloride and in the other solution 1.7 g silver nitrate were added. These two solutions are then poured one after the other into the initially prepared solution with the addition of 0.5 g of NaBH_4_. Afterwards, the final solution was stirred at 100 °C for 4 h to make it homogeneous and later centrifuged at 6000 rpm for complete extraction of NPs. The obtained bimetallic NPs were dried at 100 °C for complete elimination of moisture and impurities, to make sure that the prepared bimetallic products were in pure form [12]. Similarly, four samples with various Ag concentrations (mol 2.5%, 5%, 7.5%, and 10%) were prepared with fixed Cu ratios.

### Antimicrobial activity

In vitro bactericidal potential of Cu:Ag bimetallic NPs was evaluated against pathogenic bacteria *S. aureus, E. coli* and *A. baumannii* isolates obtained from bovine mastitic milk using well diffusion method. Mannitol salt agar, MacConkey agar and Lauria Bertani agar were swabbed with isolated bacteria activated growth 1.5 × 10^8^ CFU/ml. After media solidification, five wells were prepared using yellow pipette possessing dimensions of 15 mm diameter and ten microliter (5 µg/mL). Freshly prepared Cu:Ag bimetallic NPs were loaded into wells with different ratios in comparison with ten microliters of amoxicillin (5 µg/mL) as positive control and 50 μl of DIW as negative control. The bactericidal activity of synthesized Cu:Ag bimetallic NPs was determined by measuring inhibition zones (in mm) formed after incubation for 15 h at 37 °C.

### Statistical analysis

The bactericidal activity of synthesized NPs with inhibition zone (mm) measurements was considered statistically significant using SPSS 20.0, one-way analysis of variance (ANOVA) [57].

### Molecular docking study

**Fig. 2:**
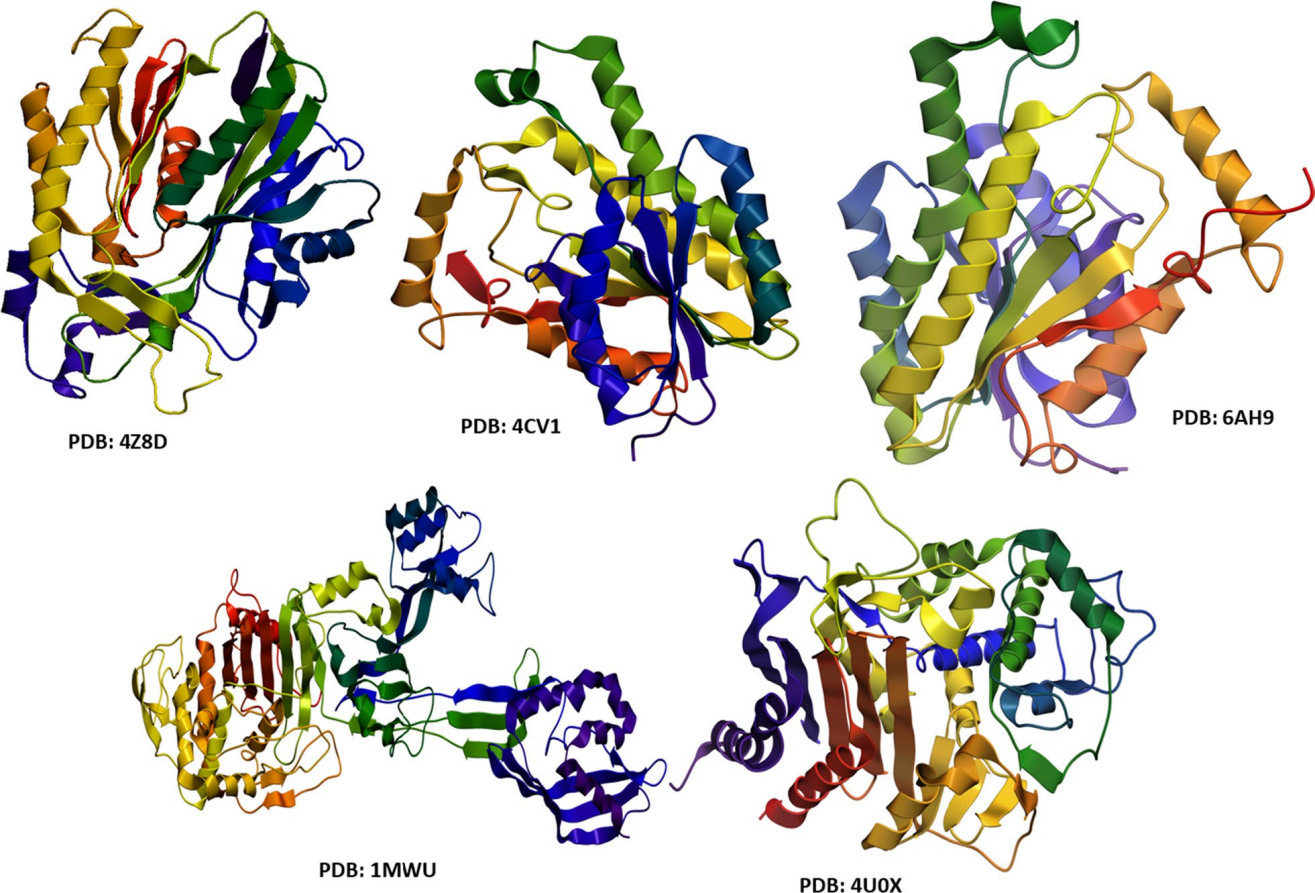
3D-structure of protein targets *β*-lactamase, FabI (from *A. bauminnii* & *S. aureus*) and FabH from *E. coli*

Antibiotics decrease bacterial growth and cause death of bacteria through cell wall damage, disrupting biochemical processes, cell membrane damage, and penetration through biofilm [38]. In recent decades, plenty of nanoparticles with potential bactericidal activity have been reported, which kill bacteria either through cell wall disruption or by blocking the food source by a mechanism similar to known antibiotics [39–41]. Hence, enzymes belonging to these biochemical pathways are thought to be an important and attractive target for antibiotic discovery [12]. Here, key enzymes from cell wall biosynthetic pathway (i.e. *β*-lactamase) and fatty acid biosynthetic pathway (i.e. FabH and FabI) were selected as possible targets to evaluate the mechanism of interaction of Cu:Ag bimetallic NPs with their active pocket as inhibitors (see Fig. 2).

3D structural parameters of selected enzymes were fetched from protein data bank with PDB code: 4U0X (2.03 Å Resolution) for *β*-lactamase [42] and 6AH9; Resolution 1.74 Å [43] for Enoyl-[acyl-carrier-protein] reductase (FabI) from *A. bauminnii*. The *β*-lactamase (3D structure) with PDB ID: 1MWU; Resolution 2.6 Å [44] and FabI with PDB code: 4CV1; Resolution 1.95 [45] from *S. aureus* while for FabH from *E. coli* has PDB code: 4Z8D; Resolution 2.0 Å [46].

Molecular docking study of Cu:Ag bimetallic NPs was performed using ICM Molsoft v3.8–4a or above (Molsoft L.L.C., La Jolla, CA) software to identify binding interactions with key residues of active site [47]. The protein/receptor preparation tool of ICM was used for optimization and structure preparation of selected enzyme targets. Steps involved were addition of polar H-atoms, deletion of water molecules, and energy minimization using default parameters. The co-crystallized ligand molecule was removed to provide room for docking of NPs. The binding pocket was defined using grid box specifying position of crystallized ligand. The conformation with lowest binding energy out of top 10 docked conformations was selected in each case to analyze interaction pattern and binding tendency of Ag–Cu bimetallic NPs inside active pocket. Pymol and discovery studio visualizer software were employed for analysis and 3D-view depiction of binding interactions [48]. The structure of Cu:Ag bimetallic NPs was retrieved from PubChem in.sdf format.

### Characterization

Structural analysis and phase purity were observed by engaging XRD (PAN analytical X’pert pro XRD) with Cu-Kα radiation (*λ* = 0.154 nm, 20° to 80°). The presence of vibrational modes corresponding to various functional groups was evaluated using Fourier transform infrared spectroscopy-FTIR with Perkin Elmer spectrometer. Absorption spectra were acquired using a UV–visible-Genesys 10S spectrophotometer. FESEM coupled with EDS spectrometer (JSM-6610LV) and HR-TEM (JEOL JEM 2100F) were employed to visualize surface morphologies. The ICM v3.8-4a or above (Molsoft L.L.C., La Jolla, CA) software was used for molecular docking analysis.

## Results and Discussion

**Fig. 3:**
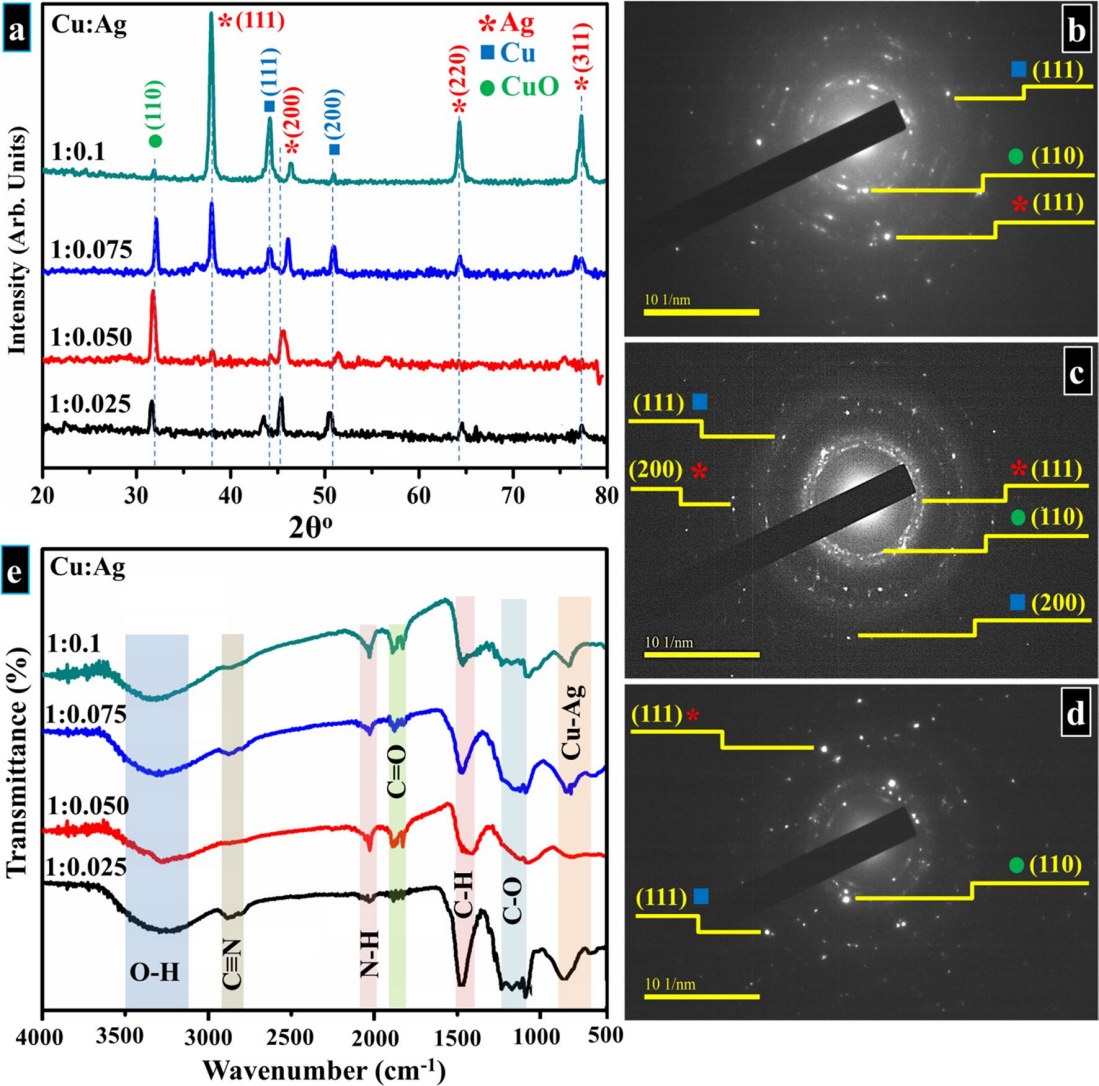
**a** XRD profiles obtained from Cu:Ag bimetallic NPs, **b**–**d** SAED rings obtained using HR-TEM for samples **b** 1:0.025, **c** 1:0.050, and **d** 1:0.10, **e** FTIR spectra of prepared samples

XRD analysis was undertaken to assess the constitution of phases and crystal structure of formulated products. Figure 3a reveals the XRD pattern plotted between 20° and 80°. In bimetallic Cu:Ag, observed reflections around ~ 38.2°, 46°, 64.4°, and 77.1° are attributed to (111), (200), (220), and (311) facets of fcc Ag phase according to JCPDS No. 04-0783 [32, 49–52]. Whereas, in the case of Cu, diffractions appearing at 32.6°, 44.2°, and 51° denoted (110), (111), and (200) lattice planes that confirmed the presence of fcc structured CuO and metallic Cu, respectively, and well-matched with JCPDS No. 04-0836 [32, 53–56]. In extracted pattern, both Ag and Cu peaks were observed which signifies the presence of NPs constituting both Ag and Cu phases. Moreover, the existence of CuO in samples with lower Ag content (e.g., 1:0.025, 1:0.050, and 1:0.075) reveals that Cu NPs were oxidized and exhibited non-protective behavior at high-temperature due to lower Ag concentrations [52]. Conversely, in the sample with the highest Ag content (1:0.1), CuO peak exhibits low intensity which indicates the formation of partially oxide-free product [57]. This suggests that improved oxidation resistance of bimetallic NPs will occur due to the addition of Ag [52]. No additional peak of impurity was detected within the instrument detection limits while each crystallographic plane comprises energetically distinct sites based on atom density. Both Cu and Ag NPs have high atom density facets at (111) that served to expose the maximum orientation of planes [51, 58]. Using Bragg’s law, d-spacing of Ag and Cu were found to be 0.24 and 0.21 nm, respectively which corresponds to distinct plane (111) of both elements and was in line with HR-TEM findings (Fig. 6) [51, 59–62]. Corresponding SAED rings (Fig. 3b–d) obtained from prepared bimetallic products display distinct ring patterns that demonstrate well-crystallized products and accord well with XRD patterns.

FTIR spectra were recorded between 500 and 4000 cm^−1^ as presented in Fig. 3e; the transmitted band positioned between 600 and 900 cm^−1^ is caused by the formation of Cu:Ag bonding [63]. The observed band around ~ 1200 and 1400 cm^−1^ is attributed to C–O and C–H, respectively; peaks appearing at ~ 1800 and 2100 cm^−1^ corresponds to C=O and N–H bonding due to PVP and NH_4_OH [64]. Transmittances observed around ~ 2800 cm^−1^ and 3400 cm^−1^ are ascribed to the presence of C≡N and hydroxyl group (O–H) [64].

**Fig. 4:**
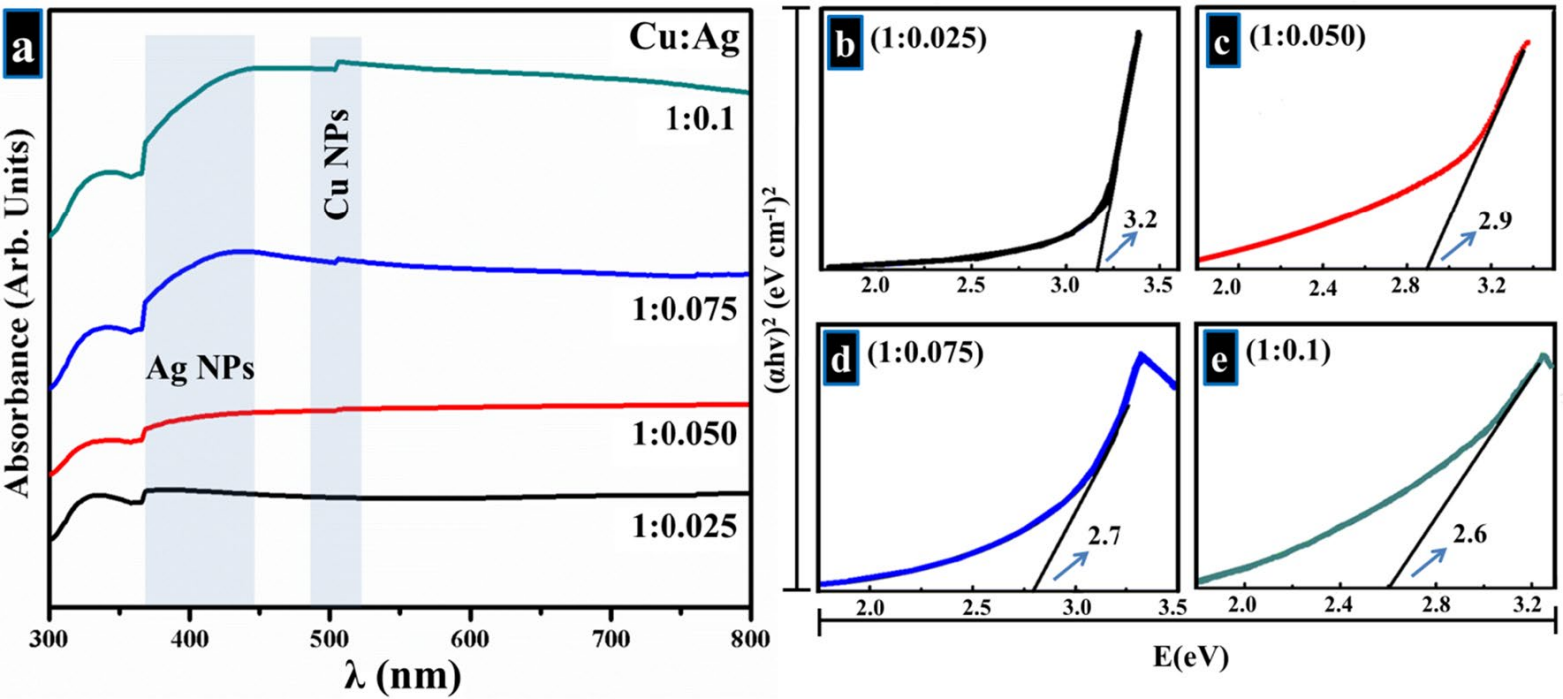
UV–Vis spectra obtained from bimetallic **b**–**e** Tauc plot analysis

Figure 4a shows the absorption spectra of Ag–Cu bimetallic NPs with clear absorption bands sited at 340, 410, and 500 nm, which are ascribed to surface plasmonic resonance absorption of metallic Ag and Cu [52]. The band appearing at 410 nm typically arises due to the presence of Ag NPs, and latter peak positioned at 510 nm is attributed to the existence of Cu NPs [52, 57–59, 65–67]. It may be suggested that bimetallic NPs are developed with distinct Ag and Cu phases, instead of bimetallic development that was also affirmed by XRD results as discussed earlier [52]. Slight redshift in absorption band at 410 nm and an increase in maximum absorption with increasing Ag content was observed [58]. Using the Tauc equation,

1 $$[\alpha h\nu = K\left( {h\nu - Eg} \right)^{n} ]$$

where *α* is considered as coefficient of absorption [2.303 log(*T*/*d*), *T* is transmitted light and *d* shows thickness of sample cell], *h* symbolizes Planck’s constant (6.62607015 × 10^−34^ Js), $\nu$ is frequency of light, *K* shows absorption index, and *E*_g_ is equal to band gap energy in eV. The value of “*n*” is related to electronic transition type of band gap [13, 26, 68, 69]. The band gap of prepared bimetallic products was calculated and found to be 3.2, 2.9, 2.7, and 2.6 eV, as demonstrated in Fig. 4b–e.

**Fig. 5:**
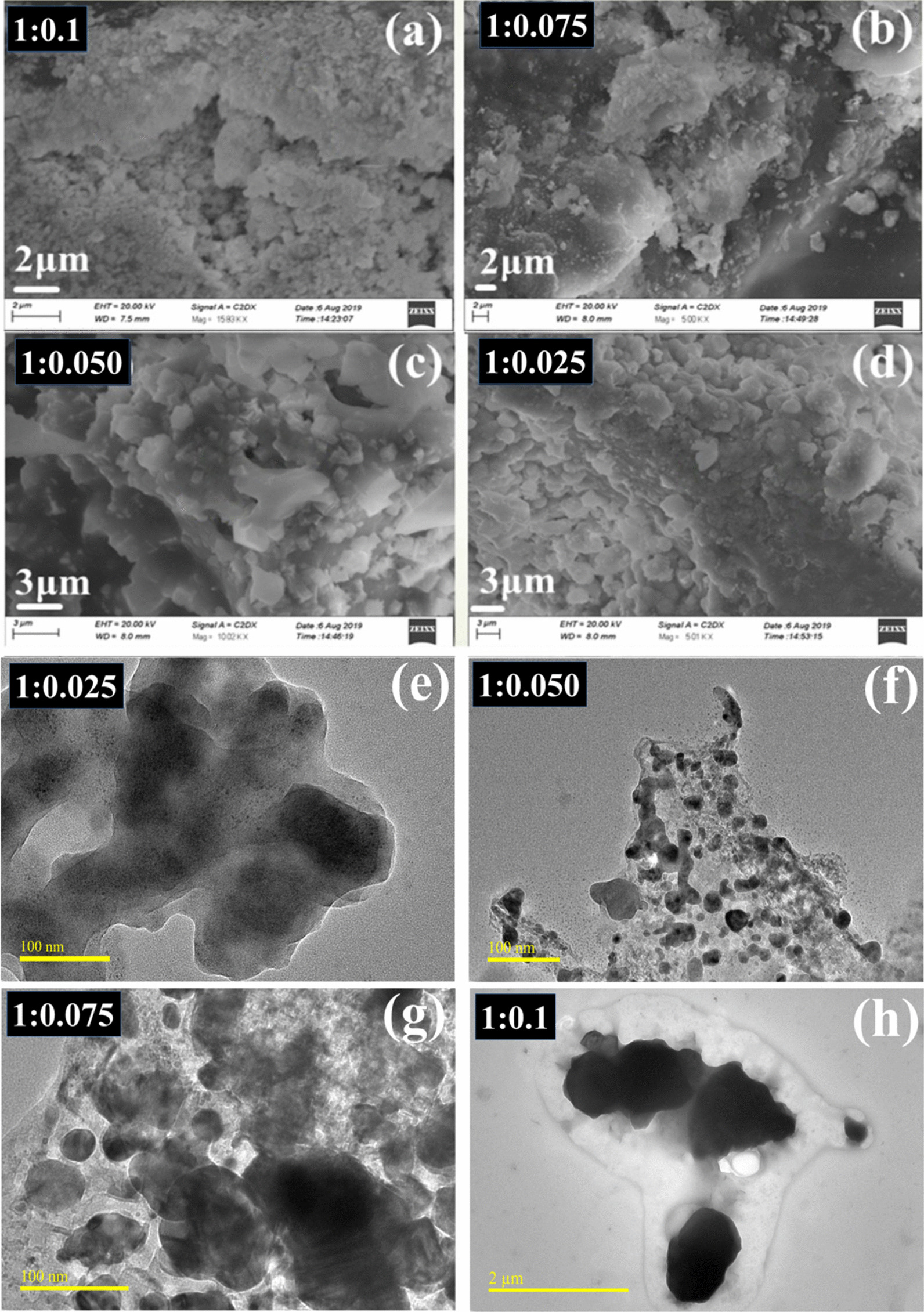
**a–d** SEM images obtained from prepared products, **e–h** HR-TEM micrographs

From SEM images (Fig. 5a–d) of as-synthesized Cu:Ag bimetallic nanocomposites, it was observed that small-sized particles were deposited on the surface of large particles. An increase in Ag content from 2.5 to 7.5% led to the formation of various particles with varying morphology which finally culminated into chunky Cu:Ag NPs. Further, accumulation of uneven and tiny Ag particles was augmented with increasing dopant concentration suggesting appearance of more scattered blocks on its surface. This signifies the major influence that Ag doping into Cu has on morphology, which was further confirmed with HR-TEM micrographs (see Fig. 5e–h).

**Fig. 6:**
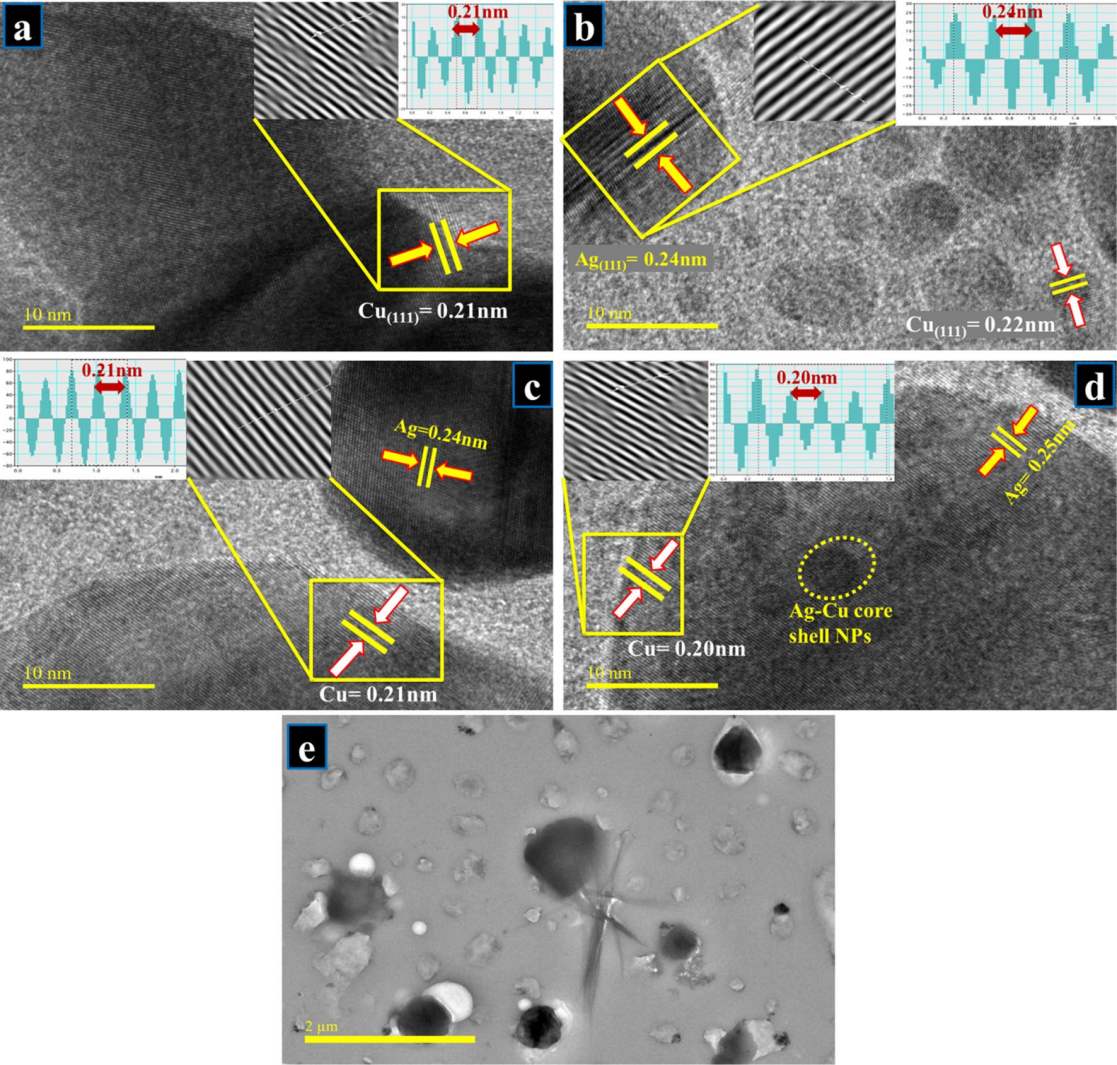
**a–d** HR-TEM (10 nm) images for d-spacing measurement for all prepared samples, **e** low magnification image showing bimetallic particles

To further elaborate on the morphology and d-spacing of prepared bimetallic, HR-TEM with 10 nm resolution was engaged. In Fig. 6a, d-spacing (0.21 nm) of Cu NPs corresponds to (111) facet of Cu, as also evident in XRD results (Fig. 3a). Figure 6b portrays a slight increase in layer spacing (0.21 to 0.22 nm) and shows Ag NPs with 0.24 nm interplanar distance that matched with (111) plane. Similarly, Fig. 6c, d shows calculated layer spacings and separate phases of bimetallic while Fig. 6e demonstrates particle shape of Ag and Cu NPs. The particles in HR-TEM images are seen to possess a core–shell structure. In Fig. 6d within a single particle, lattice fringes emanating from Cu and Ag were recorded. This strongly suggests the formation of core–shell bimetallic NPs with different Cu:Ag ratios yielding irregular quasi-spherical NPs. Furthermore, TEM image showed particles seem like as dark and bright region. This variation in contrast within a single particle may indicate the presence of two distinct constituent materials suggesting the creation of bimetallic Cu:Ag particles [70, 71].

**Fig. 7:**
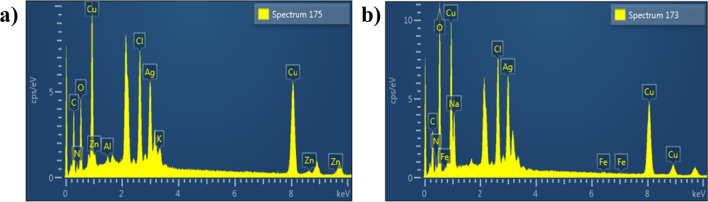
EDS profiles obtained from bimetallic NPs

The presence of distinct peak in EDS spectrum and elemental composition originating from bimetallic affirmed successful formation of Cu:Ag NPs. Figure 7a represents EDS spectrum obtained from 1:0.050 sample which shows clear peaks of Cu and Ag. Figure 7b is taken from 1:010 sample where peaks for C and O were detected in doped samples. These show up since carbon tabs are utilized to hold samples during SEM examination and/or due to background counts in SEM–EDS sensor.

In vitro bactericidal efficacy of Cu:Ag bimetallic NPs using agar well diffusion assay is presented in Table 1. The results demonstrate direct proportional relationship between synthesized NPs concentration and inhibition zones (mm). Statistically significant (*P* < 0.05) inhibition zones recorded for (2.5, 5, 7.5 and 10 wt%) Ag dopant against *S. aureus, E. coli* and *A. baumannii* ranged from 0.85–2.8 mm, 0.55–1.95 mm and 0.65–1.85 mm, respectively, see Table 1. All results were compared with DIW (0 mm) and amoxicillin (4 mm) as negative and positive control, respectively. Similarly, %age efficacy of doped NPs increased (21.2–70%), (13.7–48.7%) and (16.2–46.2%) against *S. aureus, E. coli* and *A. baumannii*, respectively. Overall Cu:Ag bimetallic NPs were found to be more potent against *S. aureus* (i.e. gram + ive) compared with *E. coli* and *A. baumannii* (i.e. gram −ive).

**Table 1:** Bactericidal action of Cu-Ag bimetallic NPs

Sample	Inhibition zone (mm)^a^	Inhibition zone (mm)^b^	Inhibition zone (mm)^c^	Ampicillin	DIW
	0.005 mg/ml	0.005 mg/ml	0.005 mg/ml	0.005 mg/ml	
1:2.5%	0.85	0.55	0.65	4	0
1:5%	1.45	0.95	1.05	4	0
1:7.5%	2.05	1.45	1.5	4	0
1:10%	2.8	1.95	1.85	4	0

^a^Inhibition zone (mm) of Cu:Ag bimetallic NPs for *S. aureus*

^b^Cu:Ag bimetallic NPs inhibition zones measurements for *E. coli*

^c^Bimetallic NPs zones of inhibition (mm) for *A. baumannii*

Size, concentration, and shape of NPs directly affect oxidative stress produced by nanostructures. Bactericidal efficacy in the form of inhibition zones (mm) improved due to greater wt% doping of Ag-doped Cu bimetallic NPs due to increased cations (++) availability. Bactericidal action in regard to size and concentration depicts an inverse relationship to size [58, 59]. Nano-sized structures produce reactive oxygen species (ROS) efficiently which reside in bacterial cell membranes leading to extrusion of cell organelles and ultimately death of bacteria [60]. Besides ROS production, cationic interaction of Ag^+^ and Cu^++^ with negatively charged parts of bacteria cell membrane results in improved bactericidal efficacy at increasing concentrations through cell lysis and bacteria collapse [58, 61].

Biological applications of various classes of nanoparticles have been extensively studied since the last few decades. Owing to unique characteristics of NPs, they have been widely utilized for their potential as bactericidal agent with the ability to substitute traditional antibiotics. NPs interact with bacterial cells, disrupt cell membrane permeation and destroy key metabolic pathways [72]. The specific mechanism of nanoparticle toxicity towards bacteria needs to be explored. It is believed that NPs interact with bacterial cell involving electrostatic forces, van der Waals forces or hydrophobic interactions that ultimately result in death of bacteria. Enzymes have been reported as main virulence factor involved in bacterial infection and targeting them to inhibit their activity aid in tackling the caused infection [73]. Here, molecular docking study of Cu:Ag NPs against enzyme targets of cell wall alongside fatty acid biosynthetic pathway identified binding interaction pattern of these NPs inside active pocket. Keeping in view in vitro antibacterial potential of these NPs against *A. bauminnii*, *S. aureus* and *E. coli*, the enzyme targets were selected from these microorganisms to get an insight into possible mechanism behind their bactericidal activity.

**Fig. 8:**
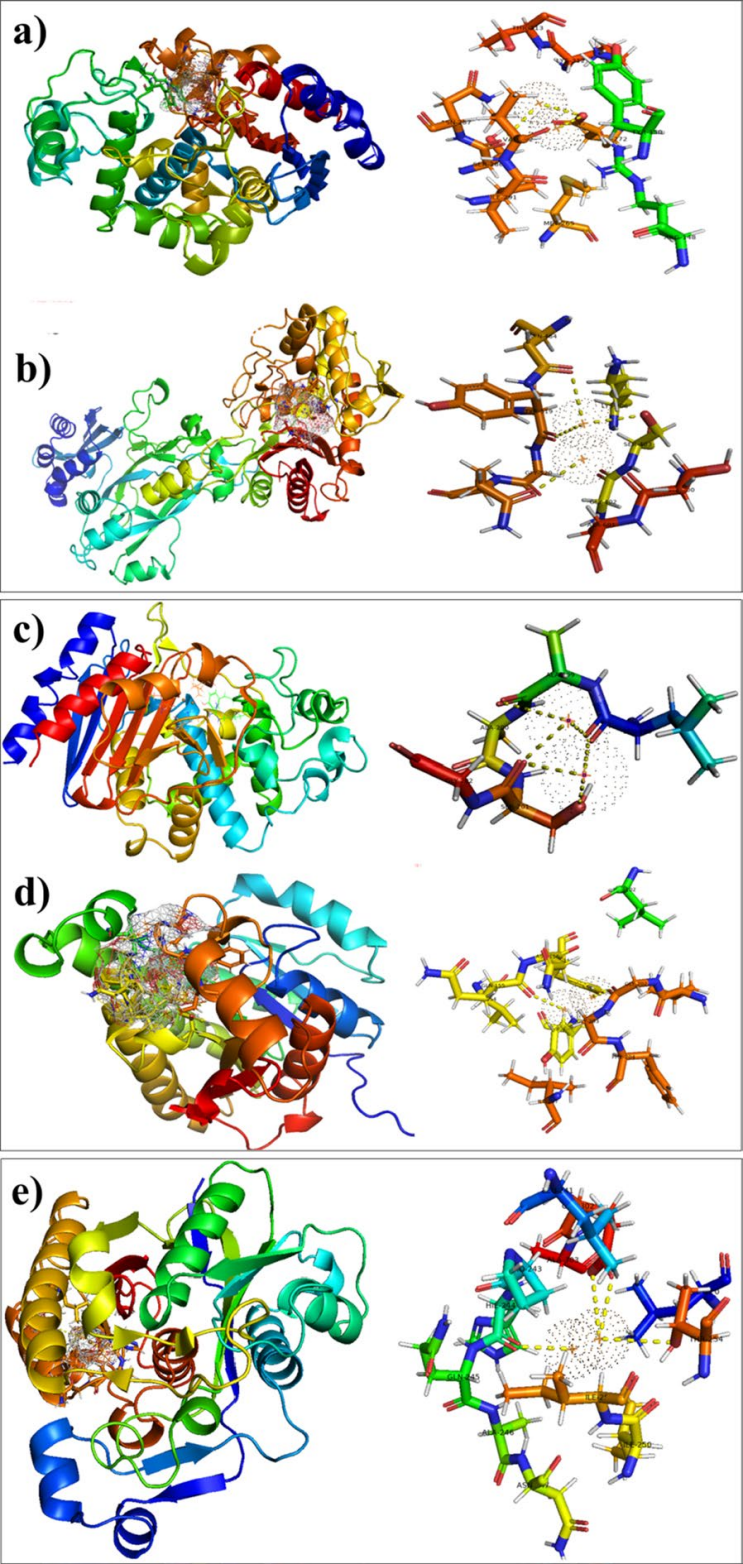
Binding interaction pattern of Ag–Cu Bimetallic NPs inside active pocket. **a** *β*-lactamase from *A. bauminnii*, **b** *β*-lactamase from *S. aureus*. **c**, **d** Binding interaction pattern of Ag–Cu Bimetallic NPs inside active pocket **c** enoyl-[acyl-carrier-protein] reductase (FabI) from *A. bauminnii*, **d** enoyl-[acyl-carrier-protein] reductase (FabI) from *S. aureus*, **e** binding interaction pattern of Ag–Cu Bimetallic NPs inside active pocket of FabH from *E. coli*

Best docked conformation observed in case of Cu:Ag Bimetallic NPs with *β*-lactamase from *A. bauminnii* revealed hydrogen bonding interaction with Glu272 (2.8 Å) and Ser286 (3.2 Å) along with metal contact interaction with Val292 while the docking score was − 4.013 kcal/mol (Fig. 8a). Similarly, binding score of Ag–Cu bimetallic NPs observed against *β*-lactamase from *S. aureus* was − 4.981 kcal/mol possessing H-bonding interaction with Ser403 (3.2 Å), Tyr519 (3.6 Å), Gln521 (3.0 Å) and Asn464 (3.1 Å) as shown in Fig. 8b.

Second enzyme target selected in the current study FabI belong to fatty acid biosynthetic pathway and molecular docking predictions suggested Cu:Ag bimetallic NPs as potential inhibitor against this target. The Cu:Ag Bimetallic NPs showed good binding score (-3.385 kcal/mol) against FabI from *A. bauminnii* having H-bonding with Ser201 (2.7 Å), Ala199 (3.5 Å), and Leu198 (3.3 Å) as depicted in Fig. 8c. Similarly, best docked conformation of Ag–Cu NPs with active site of FabI from *S. aureus* showed H-bonding with Gly202 (2.5 Å)and Gln155 (2.5 Å) having binding score − 3.012 kcal/mol (Fig. 8d).

In addition, the binding capacity of Cu:Ag bimetallic NPs against FabH from *E.coli* was also evaluated and the binding score observed was − 4.372 kcal/mol having H-bonding interaction with Thr254 (3.5 Å), HIE244 (2.6 Å) and Glu302 (3.0 Å) shown in Fig. 8e.

## Conclusion

Cu:Ag bimetallic nanoparticles were prepared through co-precipitation method for use in applications to combat bacteria-related ailments. XRD profiles confirmed the presence of fcc structured CuO and metallic Cu and Ag particles. Both Ag and Cu peaks were observed which signifies bimetallic NPs entailing Ag and Cu phases. Planes observed in XRD analysis correspond well to SAED rings. Attached chemical groups with formulated products and characteristic transmittance band between 600 and 900 cm^−1^ was caused by the formation of Cu:Ag bonding. The plotted spectra of UV–vis showed absorption at 410 nm which typically arises due to the presence of Ag NPs, and latter peak positioned at 510 nm was attributed to the existence of Cu NPs. The particles in HR-TEM images were seen to have a core–shell structure. Cu:Ag NPs clearly showed the formation of bimetallic NPs with different Cu:Ag ratios yielding irregular quasi-spherical NPs. Further, d-spacing of Cu NPs i.e., 0.21 nm corresponds to (111) facet of Cu detected in XRD results. A slight increase in layer spacing (from 0.21 to 0.22 nm) also shows that Ag NPs with 0.24 nm layer distance matched with (111) plane. Molecular docking study showed good agreement with in vitro bactericidal activity. The binding tendency of Cu:Ag bimetallic NPs against *β*-lactamase enzyme of cell wall biosynthetic pathway alongside FabI and FabH enzymes of fatty acid biosynthetic pathway demonstrated their inhibition potential that needs to be explored further through enzyme inhibition studies.

## Data Availability

All data are fully available on demand.

## Abbreviations

EDS: Energy dispersive X-ray spectroscopy
FTIR: Fourier transform infrared spectroscopy
G + ve: Gram-positive
G −ve: Gram negative
HR-TEM: High resolution transmission electron microscopy
JCPDS: Joint committee on powder diffraction standards
Ag: Silver
UV–Vis: Ultra-violet visible spectroscopy
XRD: X-ray diffraction
